# Structure and Strength of Artificial Soils Containing Monomineral Clay Fractions

**DOI:** 10.3390/ma14164688

**Published:** 2021-08-19

**Authors:** Grzegorz Jozefaciuk, Kamil Skic, Agnieszka Adamczuk, Patrycja Boguta, Krzysztof Lamorski

**Affiliations:** Institute of Agrophysics, Polish Academy of Sciences, 20-290 Lublin, Poland; k.skic@ipan.lublin.pl (K.S.); a.adamczuk@ipan.lublin.pl (A.A.); p.boguta@ipan.lublin.pl (P.B.); k.lamorski@ipan.lublin.pl (K.L.)

**Keywords:** mesostructure, mercury porosimetry, particle shape, particle dimension, clay minerals

## Abstract

Structure and strength are responsible for soil physical properties. This paper determines in a uniaxial compression test the strength of artificial soils containing different proportions of various clay-size minerals (cementing agents) and silt-size feldspar/quartz (skeletal particles). A novel empirical model relating the maximum stress and the Young’s modulus to the mineral content basing on the Langmuir-type curve was proposed. By using mercury intrusion porosimetry (MIP), bulk density (BD), and scanning electron microscopy (SEM), structural parameters influencing the strength of the soils were estimated and related to mechanical parameters. Size and shape of particles are considered as primary factors responsible for soil strength. In our experiments, the soil strength depended primarily on the location of fine particles in respect to silt grains and then, on a mineral particle size. The surface fractal dimension of mineral particles played a role of a shape parameter governing soil strength. Soils containing minerals of higher surface fractal dimensions (rougher surfaces) were more mechanically resistant. The two latter findings appear to be recognized herein for the first time.

## 1. Introduction

Soil structure is a unique and important feature governing the development of plants, absorption of nutrients, growth of roots [1], aeration and water transport [2], resistance to erosion [3], and many others features. The strength and durability of soil structure are key factors influencing a number of soil performance characteristics, such as stability of landfill liners, specific barriers for nuclear waste disposal, foundations for buildings, roads and embankments [4,5,6,7], soil bearing capacity [8,9], settlement [10], deformation by machines [11], the velocity of seismic waves [12] and many others.

Soil structure and strength are governed primarily by the soil components. Among them, organic matter, considered as the most important, has probably been studied most intensively [13,14]. A number of reports concern its effect on soil compressibility, void ratio, liquid and plastic limits [15,16,17], specific gravity, bulk density [15,16] and compressive strength [18]. The strong impact of iron oxides, alumina and silica on soil structure and strength was reported as well [19,20,21]. In general, organic matter, sesquioxides and silica decrease soil strength by increasing soil porosity and reducing bulk density.

Clay minerals, the next important factors influencing soil structure, usually increase soil strength. The extent of this effect depends on different volumetric compressibility, swelling and shrinking potential, plastic and liquid limits, density and porosity of particular minerals [22,23]. Sharma et al. [4] have reported an increase in the specific gravity, dry density, unconfined compression strength (UCS) and the Young’s modulus with an increase in bentonite proportion in bentonite-sand mixtures. Tiwari and Ajmera [24] revealed that the compression index for montmorillonite dominated soils was higher than for kaolinite or illite dominated soils. They have noticed that the intrinsic compression line was unique for each dominating clay mineral. In studies of multicomponent systems of clay minerals, Ye et al. [25] showed that montmorillonite significantly increased the compression of kaolinite-based clayey soils, while contents of illite and chlorite were less influential in these systems. The beneficial effect of bentonite on the hydraulic conductivity, mechanical stability and permeability were indicated in mixtures with sand, pond ash or fly ash [5,6,26]. Charkley et al. [27] reported an increase in cohesion and shear strength with an increase in kaolinite/montmorillonite content in artificial clay soil. Onyelowe [28] reported a significant improvement in the strength of lateritic, silty clayey soil treated with 15% of nanostructured kaolinite from 194 to 315 kPa. Rajabi and Ardakani [29] reported that the UCS of the clayey sand (20% clay) increased from 200 to 800 kPa and of the sandy clay (51% clay) from 600 to 1500 kPa after the addition of 25% zeolite. Firoozi et al. [30] observed that the addition of 50% kaolinite to silt (silica sand) resulted in an increase of UCS from practically zero to 115 kPa, whereas 50% bentonite amendment caused smaller UCS increase (to 83 kPa). Much higher increase of UCS, to 803 kPa, was reported by Sharma et al. [4] after the addition of 50% bentonite to coarser sand. However, some reports show contrasting results. Narloch et al. [7] found that in some cases montmorillonite and beidellite decreased the compressive strength of the soil. Carraro et al. [31] and Salgado et al. [32] reported that non-plastic fines, like silt, can contribute to increase both the peak and the critical-state friction angles in sand, while plastic fines, like kaolinite, can decrease these parameters. Supandi et al. [33] found that increasing content of illite in kaolinite-illite mixture added to claystone decreased cohesion, friction angle and strength from 400 kPa even to 20 kPa. Tembe et al. [34] observed that illite decreased friction coefficient in a mixture of illite and quartz. Naeini et al. [35] observed a reduction of UCS of a fine-grained clayey soil after the addition of 20% bentonite from 9.8 to 7.5 kPa.

Various results presented in the literature are often difficult to compare due to the variety of research methods and specimen shapes. Kawajiri et al. [36] reported significant differences in the strength of different soils measured by dynamic and static compaction techniques, loaded vertically or horizontally even for samples having similar grain size distribution. Horn and Fleige [37] indicated the dependence of shear forces on the preferred orientation of platy particles, suggesting anisotropy of strength properties. Ajayi et al. [38], who suggested separation of soils into different classes to enhance the reliability of predictions, also raised a similar problem. Moreover, the compression tests may give different results for the same soil, depending on sampling and, particularly, on the specimen height to diameter ratio [31,39,40,41,42]. Usually, at higher length-to-diameter ratios of the specimens the UCS values decrease. Pronounced changes in soil strength are observed with soil moisture [43], sample curing time [44] or ionic composition [45,46]. However, in general, as stated by Ye et al. [25] not much data are available concerning the effect of the four main clay minerals: kaolinite, illite, montmorillonite and chlorite, on the mechanical properties of clayey soils.

The strength of soil appears to be governed mostly by its bulk density and porosity [40,47,48,49,50,51]. However, Horn and Fleige [37] stated that this issue also requires in-depth research because the strength properties of soils may be quite different, even for similar bulk densities, due to various pore system arrangements (horizontal, vertical or randomized). The compressibility of granular materials is affected by the size [52] and the shape [53] of the grains. Sphericity, roundness, roughness and texture are some of the terms (defined in different ways) commonly used as descriptors for particle shape [54]. Since particle rotation is a basic component of deformation, as the angularity increases, the ratio of rolling to sliding contacts decreases, which leads to greater shearing resistance. Dense packing enhances the effect of angularity because as the total number of contacts between particles increases, the rotation resistance increases simultaneously [55,56]. While investigations of the influence of particle size have mainly been focused on binary mixtures of clay–sand, sand–gravel or clay–gravel, and investigations of the influence of particle shape have mainly been conducted on sands, the experiments illustrating the effects of particle shape and size distribution on the shear behavior of soils characterized by a wide range of particle sizes are rare [57].

Even from the presented short review of the existing knowledge, it can be concluded that the contribution of minerals to soil strength has not been fully recognized. This is most probably due to that soil structure and its stability are affected by dozens of different factors acting simultaneously, and so, the effects of individual minerals are hardly distinguishable. Therefore, to observe more clear mechanisms, the structure and the strength of artificial soils formed from silt fraction extracted from a loessial soil with additions of individual minerals in sodium homoionic forms (sodium has the smallest consolidation effect) were studied. Wide range of textures, from 100% silt to 100% clay were examined to find more general dependencies between strength, mineral content and structure parameters. This study also attempted to find size and shape parameters of the clay-size mineral particles which relate to the strength of the aggregates containing particular minerals, and, in consequence, to the strength of the soil. According to the authors’ knowledge, such an approach is missing in the literature published so far.

## 2. Materials and Methods

### 2.1. Substrates

Silt fraction 2–50 µm extracted from the upper 0–10 cm layer of Haplic Luvisol composed from 66% sand (2–0.02 mm), 28% silt (0.02–0.002 mm) and 6% clay (<0.002 mm), described in details by Lipiec et al. [58] was used as skeletal material. Clay particles were removed from the soil by sedimentation (4:100 solid: liquid weight by weight ratio), sedimentation time calculated for solid phase density of clay assumed to be 2.65 g·cm^−3^), while sand particles were wet-sieved out. The silt was composed mainly from feldspars and quartz.

As cementing agents smaller than 2 μm particles extracted by sedimentation (4:100 solid: liquid *w*/*w* ratio, sedimentation time calculated for the solid phase density of each mineral, separately) from powdered forms of the following minerals were used:

- goethite 71063-100G (Sigma-Aldrich, St Louis, MO, USA);
- kaolinite containing <5% illite and ~10% quartz,
- illite containing ~10% kaolinite and ~5% quartz,
- montmorillonite K10 (Sigma Aldrich Chemie GmbH, Steinheim, Germany);
- zeolite coming from a clinoptilolitic tuff deposit in Sokirnitsa, Ukraine [59] containing clinoptilolite as a dominant phase, ~10% stilbite and ~10% thomsonite.

The minerals admixtures were estimated on the X-ray diffraction spectra registered with an X’pert PRO APD MPD XRD spectrometer (PanAnalytical Philips, Almelo, the Netherlands).

Collected suspensions containing <2 μm particles of the minerals were centrifuged, the sediments were diluted with 1 bed volume of distilled water and then lyophilized. The lyophilized mineral powders were then used for the further experiments.

#### Substrates Characteristics

Material densities (solid phase densities), SPD (g·cm^−3^), were measured by helium pycnometry using an Ultrapycnometer 1000 system (Quantachrome Instruments, Boynton Beach, FL, USA) in five replicates.

Particle size distribution, PSD, expressed as a volumetric percentage of particles of a given radius in the total volume of particles, average particle diameter, *d* (m), and zeta potential, ζ (V), values were estimated at 25 °C for suspensions of 1 mg·dm^−3^ minerals in 1 dm^3^ 0.001 mol·dm^−3^ KCl solution using a ZetaSizer Nano ZS instrument (Malvern Ltd., Leamington Spa, UK) apparatus in six replicates. The PSD of the silt was estimated using a laser diffraction method using a Malvern Mastersizer 2000 according to Ryzak and Bieganowski [60]. The zeta potential of the silt was not measured with the ZetaSizer because silt particles were too large and they sedimented in the electric field. Therefore, it was taken as the average of zeta potential values for feldspar and quartz at pH = 6.5 (this pH value located between pH of the silt-mineral pastes used for aggregates preparation) read from plots presented by Wang et al. [61], who measured the zeta potential of these minerals at various pH values.

Specific surface areas, *S_N2_* (m^2^·kg^−1^), and surface fractal dimensions, *D_fracN2_*, were estimated from low temperature nitrogen adsorption isotherms, relating the adsorbed amount *a* (kg·kg^−1^) to the relative adsorbate pressure *p/p*_0_ (*p* (Pa) is the adsorbate equilibrium pressure and *p*_0_ (Pa) is the saturated pressure of the adsorbate at the temperature of the measurement T (K)). The surface areas were calculated from the standard BET equation [62] and the nanopore fractal dimensions, *D_fracN2_*, from the slopes of the linear parts of the ln-ln plots of adsorption *a* vs. adsorption potential *A* = RTln(*p*_0_/*p*), where R is a universal gas constant, using the equation [63]:

ln(*a*) = *C* − (1/*m*)ln(*A*) (1)

where *C* is a constant and the parameter *m* is related to the surface fractal dimension of the sample. The magnitude of the parameter 1/*m* distinguishes two possible adsorption regimes: when 1/*m* ≤ 1/3, the adsorption occurs within van der Waals regime and the surface fractal dimension is then *D_fracN2_* = 3(1 − 1/*m*). Alternatively, for 1/*m* > 1/3 the adsorption is governed by the capillary condensation mechanism and *D_fracN2_* = 3 − 1/*m*. The surface fractal dimension is 2 for flat, planar surfaces, and tends to 3 with increasing surface complexity and roughness.

The nanopore parameters were calculated from low temperature nitrogen desorption isotherms for pore diameters ranging between 2–30 nm. It was assumed that pores of that sizes are located mostly on the surfaces of the mineral grains and not between them. The pore radius *r* (m) was attributed to *p/p*_0_ using the Kelvin equation, assuming cylindrical shape of pores and zero solid-liquid contact angle:

log(*p/p*_0_) = 2γV_m_/*r*RT (2)

where γ is surface tension of the liquid and V_m_ is its molecular volume.

The volume of the pores at a given radius, *v*(*r*), was taken as the amount of liquid nitrogen accumulated in the material at a given (radius-corresponding) pressure, and this was treated as a sum of pore volumes, *v_i_*(*r_i_*), of the radii *r_i_* ≤ *r*:

(3) $$v(r)=\sum_{i=1}^{n}v_{i}(r_{i})$$

Dividing the above equation by the total pore volume in the region of interest, (2–30 nm nanopores), *V_poreN2_* (m^3^·kg^−1^), calculated as a difference of the volume of the liquid nitrogen accumulated in the material at a relative pressure corresponding to 30 nm pore and that corresponding to 2 nm pore), the pore size distribution function, *Ξ*(*r*), was obtained:

(4) $$\Xi \;(r)=v(r)/V_{poreN2}=\sum_{i=1}^{n}v_{i}(r_{i})/V_{poreN2}=\sum_{i=1}^{n}f(r_{i})$$

where *v*_i_(*r*_i_)/*V_poreN2_* is a volumetric fraction of particular pores, *f*(*r*_i_), calculated as:

f(*r*_i_) = f(*r*_i,av_) = *Ξ* (*r*_i+1_)−*Ξ* (*r*_i_) (5)

where *r*_i,av_ denotes the arithmetic mean of *r*_i+1_ and *r*_i_. 

The average pore radius, *r_poreN2_* (m), was calculated as:

(6) $$r_{\mathrm{av}}=\sum_{i=1}^{n}r_{i,\mathrm{av}}\;f(r_{i,\mathrm{av}})$$

The adsorption and the desorption isotherms were measured using 3Flex surface analyzer (Micromeritics Inc., Norcross, GA, USA) in three replicates.

### 2.2. Preparation of Artificial Soil Aggregates

Mixtures composed from the lyophilized mineral powders ant the silt were homogenized by 1 h shaking on a rotary shaker (100 rpm). Water-saturated pastes were made from the above mixtures. Distilled water was used for pastes preparation. A few additional drops of water were added to the pastes and the obtained cakes were homogenized again by hand-mixing with a glass piston for around 20 min. The cakes were then dried to return to a full water saturation state. The water saturation state of the pastes was assessed organoleptically. It was assumed that a slight excess of the added water will not change the structure of the finally air-dried aggregates. The content of particular minerals in the mixtures was 0, 2, 4, 8, 16, 32, 64 and 100%. The pH of the pastes was between 6.0 and 7.3. Twenty cylindrical aggregates of 10 mm diameter and 20 mm height were formed from the pastes for each mineral/silt ratio, which then were dried until constant mass at laboratory conditions (relative humidity around 60% and temperature around 23 °C).

#### The Aggregates Characteristics

Uniaxial compression tests were performed using material testing machine Lloyd LRX (Lloyd Instruments Ltd., Bognor Regis, UK). The aggregate placed vertically on the machine basement was pressed by a piston. The load measured with the accuracy of ± 0.05 N against displacement of the piston moved with the rate of 10^−5^ m·s^−1^ was registered for ten replicates of each aggregate. The average breakage curve for each aggregate was calculated from at least six breakage curves being most similar among ten experimental replicates. Reasons of the breakage curves selection are explained in the Supplementary Materials. From the average braking curves the dependence of the compression stress, σ (MPa), (load divided by the aggregate cross section area) versus strain, ΔL/L (relative aggregate deformation, equal to piston displacement divided by the aggregate height) was calculated. From the slope of the linear parts of the above dependence the Young’s modulus, *E* (MPa), was derived. As found by Horabik and Jozefaciuk [48], for aggregates prepared from kaolinite and 10–50 µm silt, the dependence of the compressive strength at breakage, σ_max_ (MPa) (maximum compression) on mineral percentage could be satisfactorily fitted to a Langmuir-type function. The same function to describe the dependencies of both the maximum compression and the Young’s modulus on mineral percentage, *M%*, for the studied aggregates was used:

*Y* = *C*(*kM%*)/(1 + *kM%*) (7)

where *Y* is either σ_max_ or *E* and *C* and *k* are constants.

The above constants were estimated from the best fits of Equation (7) to the respective experimental data using Solver tool built in the Microsoft Excel 2003.

The bulk density (*BD*) of the aggregates were estimated for laboratory dried specimens. The aggregate mass minus the moisture content was divided by the volume of the aggregate measured by its compulsive immersion in mercury. Moisture of the aggregates was measured by weighing after overnight drying at 105 °C. Five aggregate replicates were used for *BD* measurements. Knowing the *BD* and the material densities, *SPD*, the volumes of pores inside the aggregates, *V_(BD)_* (cm^3^·g^−1^), were calculated. 

Mercury intrusion porosimetry (MIP) tests were performed for pressures ranging from c.a. 0.1 to 200 MPa (pore radii from c.a. 10.0 to 3.8·10^−3^ µm) using the Autopore IV 9500 porosimeter (Micromeritics, Norcross, GA, USA) for three replicates of each aggregate. The intrusion volumes were measured at stepwise increasing pressures allowing to equilibrate at each pressure step. The maximum deviations between the mercury intrusion volumes were not higher than 6.2% and they occurred mainly at low pressures (largest pores). The volume of mercury *V_MIP_* (m^3^·kg^−1^) intruded at a given pressure *P* (Pa) gave the pore volume that can be accessed. The intrusion pressure was translated on equivalent pore radius *R_MIP_* [m] following the Washburn [64] equation:

*P* = −*A* σ_m_ cosα_m_/*R_MIP_* (8)

where σ_m_ is the mercury surface tension, α_m_ is the mercury/solid contact angle (taken as 141.3° for all studied materials) and *A* is a shape factor (equal to 2 for the assumed capillary pores).

Knowing the dependence of *V_MIP_* vs. *R_MIP_*, a normalized pore size distribution, *χ(R_MIP_)*, was calculated and expressed in the logarithmic scale [65]:

*χ*(*R_MIP_*) = 1/*V_MIP_*_,max_ d*V_MIP_* /dlog(*R_MIP_*), (9)

where *V_MIP_*_,max_ is the maximum amount of the intruded mercury (at the highest pressure). 

The *V_MIP_*_,max_ may be treated as the total volume of material pores accessible by mercury.

Knowing χ(*R_MIP_*), the average pore radius, *R_MIPav_*, was calculated from:

*R_MIPav_* = ∫*R_MIP_ χ*(*R_MIP_*) d*R_MIP_*. (10)

The penetration thresholds, *PT* (m), into the pores inside the aggregates, i.e. the points at which mercury starts to enter the aggregates [66], were approximated by the pore radii at which the second derivative of pore volume vs. log radius equals zero [67]:

d^2^*V_MIP_*/d(log*R_MIP_*)^2^ = 0 (11)

The intrinsic pore volume within an aggregate, *V*_ia_, was taken as the volume of mercury intruded into the pores of lower radii than *PT* (at higher pressures than this corresponding to *PT*) and the average intrinsic pore radius, *R*_ia_, (radius of pores smaller than *PT*) was calculated from Equation (10) in the same way as *R_MIPav_*, placing *V*_ia_ instead of *V_MIP_*_,max_ in Equation (9).

The pore surface fractal dimension, *D_fracMIP_*, was calculated from the slope of the linear part (if any) of the dependence of log(d*V*/d*R_MIP_*) against log*R_MIP_* [68]:

*D_fracMIP_* = 2 − slope (12)

The procedure of Yokoya et al. [69] was applied to estimate linearity ranges.

Images of the surfaces of the aggregates broken by hand were taken in ten replicates using the Phenom ProX desktop SEM provided by Thermo Fisher Scientific (Waltham, MA, USA).

## 3. Results and Discussion

### 3.1. Properties of the Substrates

**Figure 1:**
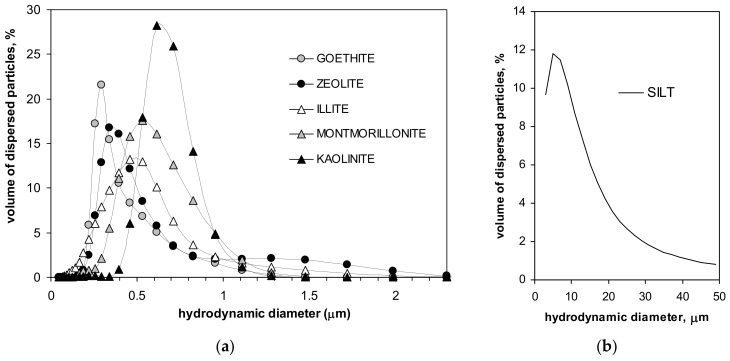
Particle size distributions of the studied minerals (**a**) and of the silt (**b**).

Particle size distributions of the minerals and of the silt are presented in Figure 1.

From the location of the peaks in particle size distributions, one can conclude that the dimension of the dominating particles in the studied minerals increases in the order: goethite, zeolite, illite, montmorillonite and kaolinite. In the silt, particles of around 10 µm dominate. Parameters characterizing the studied materials are presented in Table 1.

Among the studied minerals, montmorillonite has the highest surface area and goethite has the smallest surface area. It is worth noting that the specific surface of the colloidal-size zeolite used in the experiments is extremely low. Zeolites are usually reported to have specific surfaces exceeding one hundred square meters per gram. Grinding of the material should lead to a further increase in surface area [70]. However, Yates [71] observed that the surface area of zeolites varied from around ten to a few hundred m^2^·g^−1^ depending on a degree of crystallinity of the mineral. Possibly, the zeolite used in this paper had a low degree of crystallinity, or it decreased under long-term immersion in water during sedimentation. The extent of the surface area does not correlate with the mineral particle dimension, indicating a marked contribution of internal surfaces into the total surface of the minerals. All materials but the goethite exhibit negative surface potential at the experimental conditions. The most negative zeta potential has montmorillonite, and the least negative-illite.

Parameters presented in the last three rows of Table 1 characterize particle geometry. The surface fractal dimension, as related to surface roughness, is the first among them. The second one is the material porosity, calculated as a ratio of micropore volume (cm^3^·g^−1^) to the material volume (cm^3^·g^−1^). The material volume was calculated as a sum of micropore volume (*V_poreN2_*) and the volume of the solid phase (equal to 1/*SPD*). The third one is a ratio of the average pore diameter, *d_poreN2_*, to the average particle diameter of the material, *d_particle_*. Since the latter two values were very small, they are expressed as percents.

**Figure 2:**
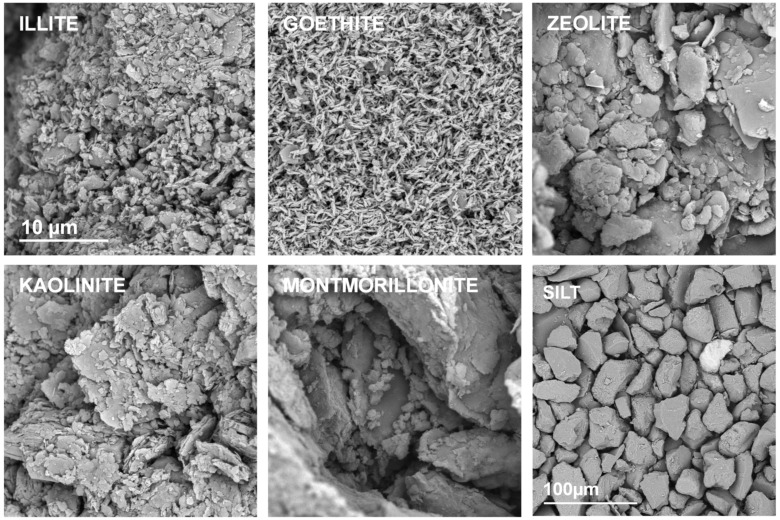
Representative SEM photographs of the surfaces of the broken aggregates composed from pure materials. The names of the materials are written within the respective pictures. The scale bar for silt is 100 µm and for all the minerals it is 10 µm (drawn only in illite picture).

Representative SEM images of the studied mineral aggregates are presented in Figure 2. In this Figure the silt aggregate composed from large feldspar/quartz grains is also presented to complete the picture.

A strong adhesion of mineral particles may be observed in SEM images of pure mineral aggregates. The individual particles can be distinguished only for goethite and possibly for illite. Kaolinite and zeolite form larger agglomerates, whereas the largest ones occur for montmorillonite.

### 3.2. Mechanical Properties of the Aggregates

**Figure 3:**
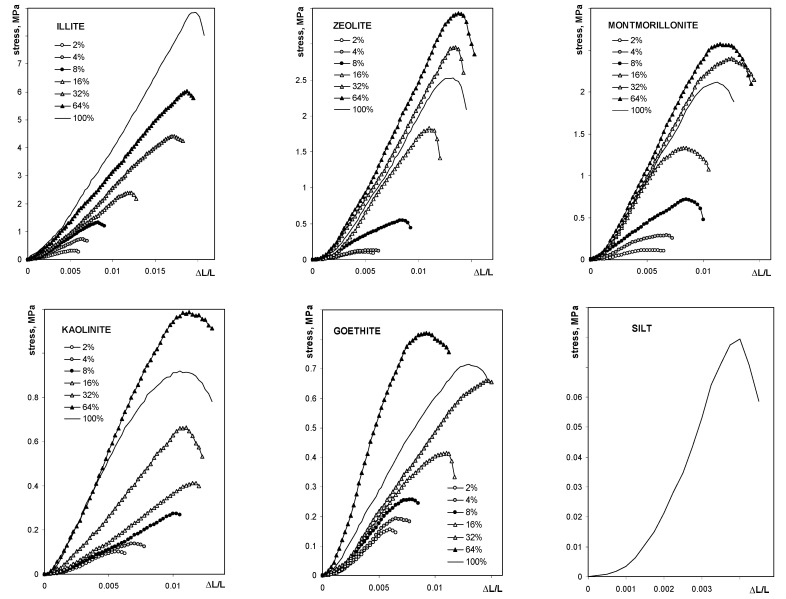
Dependence of the compressive stress on the relative deflection for the studied aggregates. The names of the materials are written within the respective pictures. Note different lengths of the units in different plots.

The average breakage curve curves for all studied aggregates are shown in Figure 3.

**Figure 4:**
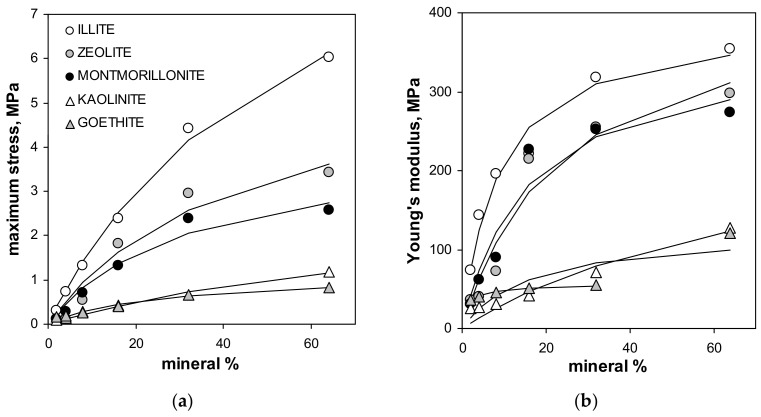
Dependencies of the maximum stress (**a**) and the Young’s modulus (**b**) of the studied aggregates on the mineral concentration. Dashed lines are the best fits of the experimental data to the Langmuir-type equation (Equation (7)). The best fit of the Young’s modulus for goethite does not include the point for 64% of this mineral (the fit including all points is drawn with solid line). The labels of the points for given mineral are the same in both plots.

The maximum tension strength for all aggregates, with exception of these containing illite, was the highest at 64% of mineral content. The maximum stress, as well as the Young’s modulus for silt-mineral aggregates (excluding pure sit and pure minerals aggregates), could be satisfactorily fitted to a Langmuir-type equation (Equation (7)), which is shown in Figure 4.

Note that the value of the Young’s modulus for the aggregate containing 64% of goethite is more than two times higher than that for the three previous mineral concentrations. Including 64% goethite into the fit gave a very weak prediction for all experimental data for this mineral by the Langmuir model, which is drawn using the solid line in Figure 4. The Langmuir model worked much better up to 32% of the goethite concentration. Therefore the 64% point was excluded from the goethite fit. The numerical values of *C* and *k* constants providing the best fits of the experimental data to the Langmuir-type equation presented in Figure 4, are shown in Table 2.

**Figure 5:**
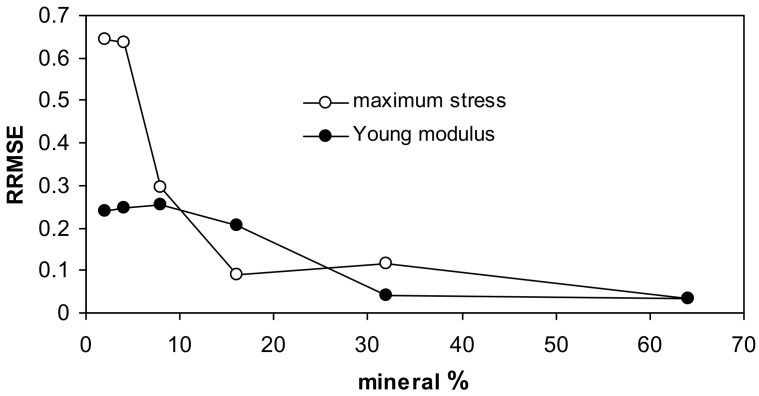
Relative root mean square errors for the approximation of the measured data by the Langmuir-type equation for all studied aggregates together.

For both fits, the highest uncertainty occurs for low mineral concentrations, as it is seen from the relative root mean square error (RRMSE) values presented in Figure 5.

The Langmuir type function may be considered as an empirical model expressing mechanical resistance of soils in relation to clay fraction percentage. Whether *C* and k constants obtained here can be applied for natural soils of different mineralogy seems worth checking.

### 3.3. Structural Properties of the Aggregates

**Figure 6:**
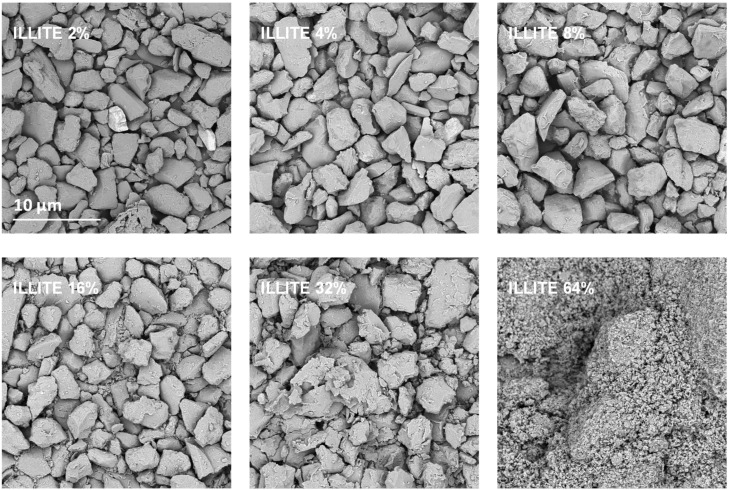
Representative SEM images of the surfaces of broken silt-illite aggregates with different concentrations of the mineral. The scale bar shown for 2% illite is valid for all other images.

**Figure 7:**
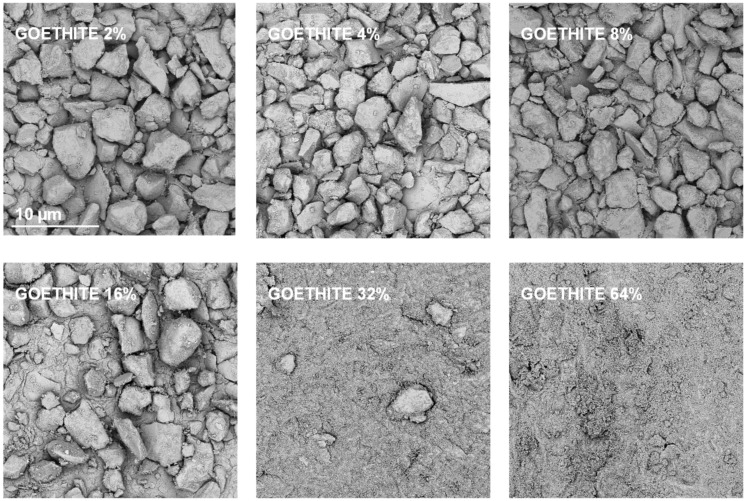
Representative SEM images of the surfaces of broken silt-goethite aggregates with different concentrations of the mineral. The scale bar shown for 2% goethite is valid for all other images.

Exemplary SEM images taken at the same magnification for the studied silt-mineral aggregates are presented in Figure 6 and Figure 7. Because illite containing aggregates had the highest, and goethite containing aggregates had the lowest mechanical durability, they were selected for the presentation.

**Figure 8:**
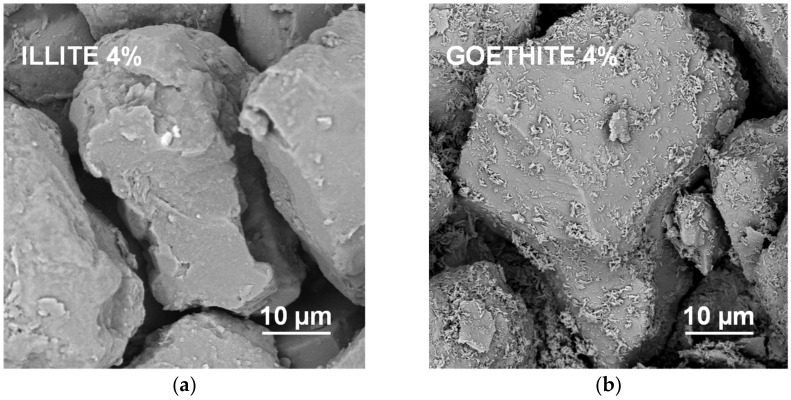
High magnification SEM images of the surfaces of aggregates containing 4% of illite (**a**) and 4% of goethite (**b**). In contrast to the illite particles, individual particles of goethite are located upon the surfaces of the silt particles.

It appears that neither particles nor agglomerates of the illite are visible at the surface of the broken aggregates up to 8% of the mineral concentration. At higher concentrations, the agglomerates of illite particles located between the silt grains are visible, and at the maximum concentration studied (64%) the silt particles are submerged in the illite phase. On the contrary, the goethite particles adhere to the surfaces of silt grains just at the lowest mineral concentrations. At higher concentrations, goethite locates both upon and between silt grains. The goethite phase completely submerges the silt particles at lower concentration (32%) than the illite (64%). Differences in location of illite and goethite particles against silt grains, are clearly visible at higher SEM magnifications presented in Figure 8.

Goethite had smaller particles than illite (see Table 1). Since the aggregate strength should be higher for smaller particles, the goethite containing aggregates should be stronger. However, they were markedly weaker than the aggregates containing all other minerals. The accumulation of goethite particles on silt surfaces may be responsible for this effect. Positively charged goethite particles could be attracted by negatively charged silt particles, at the stage of water saturated pastes during aggregates preparation. At his stage, the amount of water may be sufficient to expand electric double layer and develop attractive forces between oppositely charged particles. The spongy layer of goethite formed upon silt grains may be extremely fragile. This indicates that the mutual location of particles may play very important role in soil mechanical stability. 

**Figure 9:**
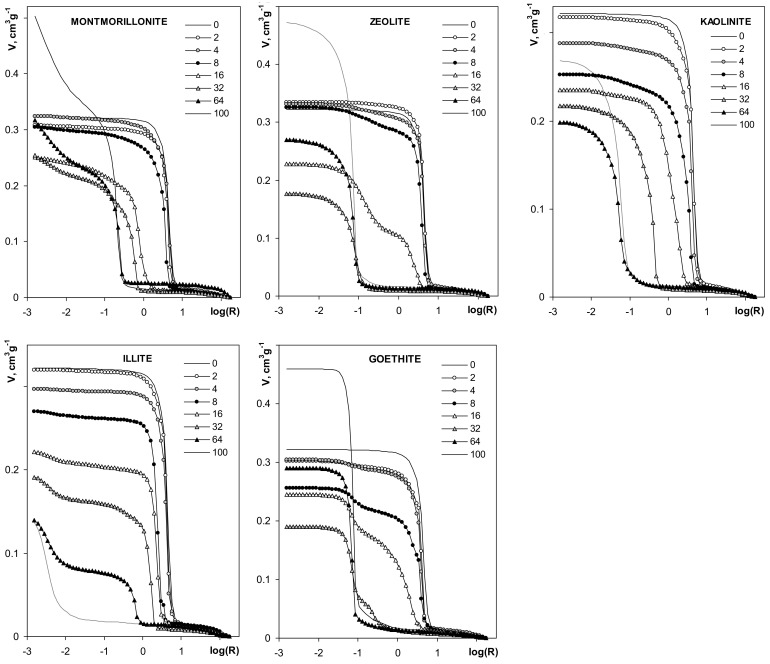
Dependencies of pore volume on pore radius derived from mercury intrusion porosimetry. The names of the materials are written within the respective pictures. Note different lengths of the y-units in different plots.

Mercury porosimetry curves of the studied aggregates are shown in Figure 9.

The volume of pores for silt-mineral aggregates is generally smaller than the pore volume of the pure silt aggregate. The pore volume of the aggregates decreases with the increase of mineral content, in the range of 2–32% mineral concentrations. The volume of pores at 64% mineral content decreases further for illite and kaolinite, and it increases for the other minerals. The pore volumes of pure montmorillonite, zeolite and goethite aggregates are higher than the pore volume of the pure silt aggregate. The pore volume of pure kaolinite aggregates is lower than for pure silt, and higher than for the aggregates containing more than 4% of this mineral. The pore volume of pure illite aggregate is the smallest within the whole aggregates population.

**Figure 10:**
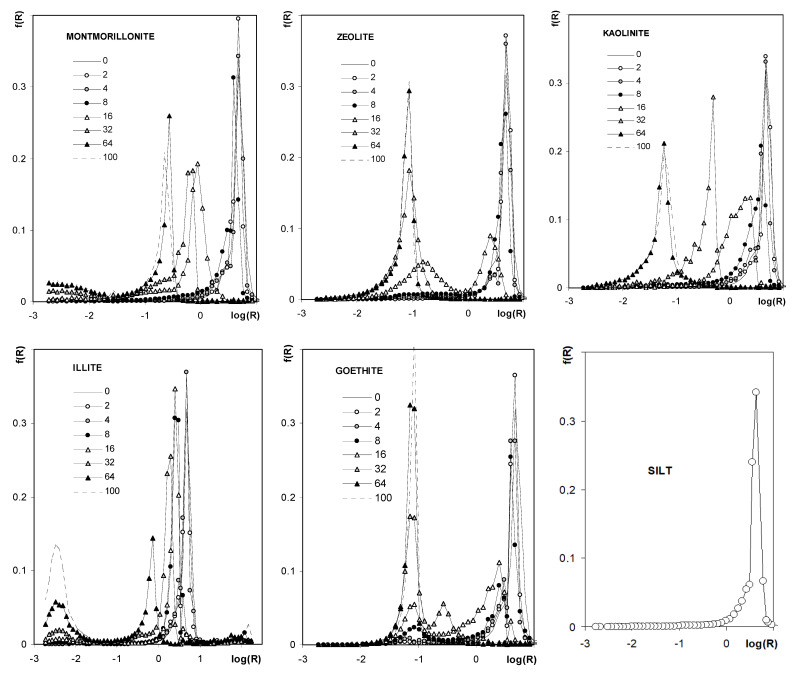
Pore size distribution functions showing frequency, f(R), of the occurrence of pores of various radii, R. The names of the materials are written within the respective plots. Since curves for 0% of each mineral are hardly visible in the respective plots, the common curve (SILT) is depicted also (0% of each mineral = pure silt).

Figure 10 shows the pore size distribution functions (PSD) calculated from the curves presented in Figure 9. Pore size distribution functions for silt and all aggregates of pure minerals are unimodal. The unimodal character of the distribution function occurs also for aggregates containing kaolinite and montmorillonite. For the latter aggregates, the dominant pore peak shifts towards smaller pore sizes with an increase in mineral concentration. Pore distribution functions are bimodal for aggregates containing the other minerals, for which peaks of larger pores also shift towards smaller radii with an increase in mineral concentration, and, simultaneously, peaks located at small pore radii (the same as for pure minerals) increase.

### 3.4. Relations between Mechanical and Structural Properties of the Aggregates

The numerical values of parameters characterizing the mechanical and structural properties of the studied aggregates are summarized in Table 3. 

It is necessary to mention that the calculated fractal dimensions of the pore surfaces are larger than 3 for most of the studied aggregates. This may result from the specific structure of the aggregates. The large pore voids are accessible through markedly narrower entrances, therefore, the volume of mercury forced into a large pore is attributed to the radius of the entrance and not to the radius of the void. In fractal dimension calculations a cylindrical pore model was applied assuming that the pore is a cylindrical capillary having the radius equal to the radius of its entrance. Attributing high void volume V to a cylindrical pore radius R the cylindrical pore model calculates high dV/dR values that gives fractal dimensions higher than 3 [72]. Nevertheless, these high “fractal dimensions” may be considered as useful structural parameters.

**Figure 11:**
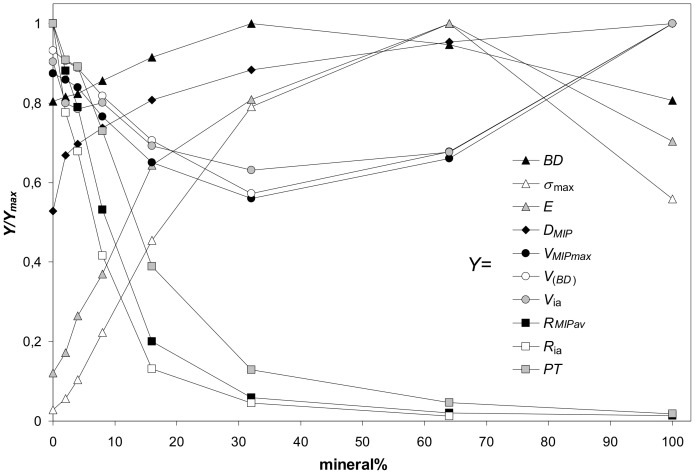
Dependence of the scaled values of parameters characterizing the studied aggregates (*Y/Y_max_*) on mineral percentage. Here *Y* is a value of a particular parameter at a given mineral content and *Y_max_* is the maximum value of this parameter. The parameters *Y* (listed in the legend) are: *BD*—bulk density, *σ_max_*—maximum stress, *E*—Young’s modulus, *D_mip_*—pore surface fractal dimension, *V_MIP,_*_max_—pore volume (from mercury intrusion), *V_(BD)_*—pore volume (from bulk density), *V*_ia_—intraaggregate pore volume, *R_MIP av_*—average pore radius (from MIP), *R*_ia_—intraaggregate pore radius, *PT*—penetration threshold. The *Y/Y_max_* values are averages for all aggregates. 100% illite aggregate is excluded from the calculation of the average Young’s modulus and average maximum stress.

As far as the parameters characterizing aggregates of various minerals collected in Table 3 undergo similar dependencies against mineral percentage, their general trends are illustrated basing on the average values of each parameter. Since the numerical values of particular parameters differ markedly, each parameter (named *Y* for this purpose) was divided by its maximum value (*Y_max_*) to present everything in a single figure. The dependencies of the above defined scaled values (*Y/Y_max_*) of parameters characterizing the studied aggregates on mineral percentage are shown in Figure 11. Because the maximum strength and the Young’s modulus of 100% illite aggregates did not follow the general trends, these values were not taken to the calculation of the average values of the respective two parameters. A reason of such anomalous behavior of illite is for us not clear. Possibly in the cake and in the aggregates of platy illite particles the input of face-to-face blocky structures prevails over edge-to face (cardhouse) ones. The blocky structures resist the pressure more strongly than the cardhouse ones.

In general, the maximum strength and the Young’s modulus of the aggregates (illite is the exception, see Figure 3) were the highest at 64% of minerals content. Similar observations were reported by Charkley et al. [27], who observed maximum strength of quartz mixtures with kaolinite and bentonite at 70% minerals content (i.e. samples with 60 and 80% minerals were weaker). As seen from SEM images, at these concentrations the silt particles appear to be completely submerged in the mineral phases which have generally lower mechanical resistance (except for 100% illite phase). Therefore, the mechanical resistance of 64% mineral aggregates should be similar to the aggregates of pure minerals.

It appeared logical that the maximum mechanical resistance of the aggregates should occur at the moment when all pores within the silt skeleton become filled with the minerals. At this moment, the maximum possible bulk density of the aggregates, *BD_EXT_*, should be reached, as well. Assuming that the minerals fill the silt pores as agglomerates which have the same bulk density as pure (100%) mineral aggregates, the *BD_EXT_* and the percentage of the mineral at *BD_EXT_*, *M%*_(*BD EXT*)_, were calculated. The *M%*_(*BD EXT*)_ values are: 35.3% (kaolinite), 28.2% (montmorillonite), 28.1% (zeolite), 39.8% (illite) and 33.3% (goethite). All these values are markedly lower than 64%, at which the maximum shear strength occurred. The theoretical maximal bulk densities at the moment when all silt pores are filled by the mineral agglomerates, B*D_EXT_*, are: 2.13 (kaolinite), 1.92 (montmorillonite), 1.91 (zeolite), 2.29 (illite) and 2.06 g·cm^−3^ (goethite). All these values are markedly higher than bulk densities of particular aggregates measured at all mineral concentrations. Since the above considerations are valid only when the silt skeleton structure remains unaltered, one can conclude that quite different structures are formed during silt-minerals aggregation processes. Most probably direct contacts between the skeleton grains disappear because the mineral particles push them away from each other. It is also possible that the structure of mineral agglomerates joining the silt grains is different from the structure of pure mineral aggregates.

The soil strength is considered to be proportional to the bulk density. In our experiments, the maximum bulk density of the aggregates was achieved generally at 32% of minerals content, markedly earlier than the maximum stress. The reason for it remains a question for us. Possibly it may be an occasional occurrence caused by rather large distance from 32% to 64%. Somewhere within this range the values of *BD* and maximum stress may coincide better.

Parameters characterizing the pore volume (the pore volume calculated from bulk density, the total pore volume measured from MIP, and the intraaggregate pore volume) change opposite to bulk density: they decrease with mineral content reaching a minimum at 32% of the mineral, and next, increase. The parameters characterizing aggregate pore sizes (the average pore radius, the intraaggregate pore radius and the penetration threshold) decrease sharply with the increasing mineral percentage, reflecting the input of fine pores present in the mineral agglomerates.

**Figure 12:**
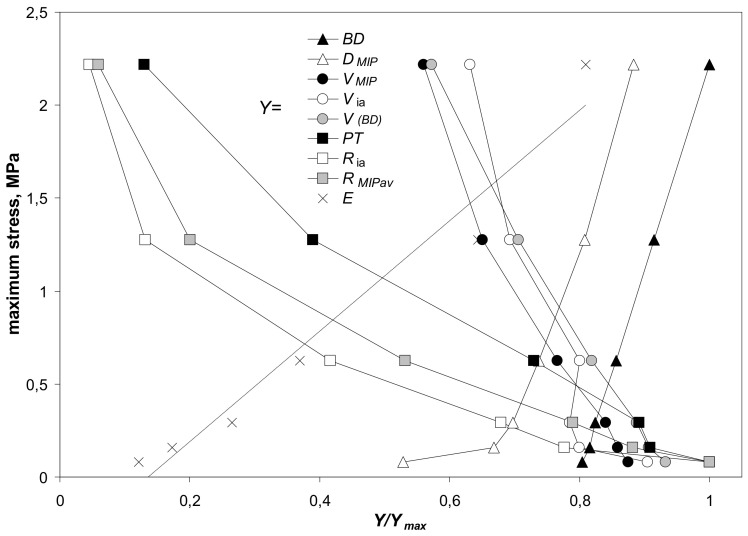
Dependence of the maximum stress on the scaled values, *Y/Y_max_*, of parameters characterizing the studied silt-minerals aggregates containing 2–64% of the minerals. Here *Y* is a value of a particular parameter at a given mineral content and *Y_max_* is the maximum value of this parameter. The parameters *Y* in the legend from top to bottom are: *BD*—bulk density, *D_MIP_*—pore surface fractal dimension, *V_MIP,_*_max_—pore volume (from mercury intrusion), *V*_ia_—intraaggregate pore volume, *V_(BD)_*—pore volume (from bulk density), *PT*—penetration threshold, *R*_ia_—intraaggregate pore radius, *R_MIP av_*—average pore radius (from MIP), *E*—Young’s modulus. The *Y/Y_max_* and maximum stress values are averages for all aggregates.

The impact of the aggregates’ parameters on their strength (maximum stress) is illustrated in Figure 12. This figure is drawn only for silt-mineral aggregates containing 2–64% of the minerals. Similar picture was obtained for the Young’s modulus (not presented because the Young’s modulus is, roughly, proportional to the maximum stress).

In general, the mechanical resistance of the studied aggregates (expressed by the maximum stress) is dependent on aggregate structural properties. It linearly increases with the increase in bulk density. Weaker aggregates have higher porosities and bigger pore radii that may be coupled with changes in bulk density. The mechanical resistance increases also with an increase in pore complicacy, determined by pore surface fractal dimension.

### 3.5. Relations between Mechanical Properties of the Aggregates and Properties of the Mineral Particles

Our trials to find relations between parameters characterizing aggregates strength and properties of mineral particles for all studied minerals failed because of anomalous behavior of goethite containing aggregates, particularly due to that they were weakest despite that the goethite particles were the smallest (as shown before, the mechanism of binding of goethite particles with the silt ones was quite different than for other minerals). Therefore, the mentioned parameters were correlated only for aggregates containing the other minerals. The results are shown in Table 4.

It can be observed that with the decrease in particle diameter the strength of the aggregates increases. This generally accepted rule appears to be valid also for clay-size particles. The strength of the aggregates increases also with an increasing fractal dimension of the particle surface. If the surface is rougher, the internal friction is higher and the rolling of the particles at the external load is retarded. Using an analogy with the Langmuir equation, k*_σ_* constant in the Langmuir-type fit of the maximum stress against mineral percentage may be interpreted in terms of particle binding energy, as mentioned by Horabik and Jozefaciuk [48]. One can say that particle binding energy increases with specific surface, pore volume and porosity of the mineral particles. It seems rational because the latter parameters govern the amount of water adsorbed on the surface at low moistures (the aggregates were studied at low relative water pressures) and the cohesion forces between particles depend on the amount of water. The energy of particle binding seems to decrease with increasing zeta potential that appears to be connected with particles repulsion. However, the electric repulsion forces should have no significant role at low moistures where the electric double layer is suppressed to a great extent and the electric charge of particles is screened by a tight layer of counterions. As can be read from adsorption isotherms of various minerals presented by Jozefaciuk and Bowanko [73] the minerals hold around two monomolecular layers of water molecules at 60% moisture, at which our aggregates were studied. The electric repulsion should be more pronounced at higher moistures.

## 4. Conclusions

The effect of minerals addition on the mechanical strength of silt aggregates increased in the order illite > montmorillonite > zeolite > kaolinite > goethite. Goethite, having the smallest particles, had the smallest effect on aggregate strength because its particles located on the silt surfaces form fragile, spongy structures. The other minerals seemed to locate in internal spaces between silt grains. 

The Langmiur-type function was used as an empirical model relating both shear strength and the Young’s modulus to the mineral percentage. It was well-fitted to the measured data for illite, kaolinite, montmorillonite and zeolite in the concentration range from 1 to 64% and for goethite in 1–32% range.

Structural properties of aggregates governed their mechanical resistance. The latter increased with bulk density and complexity of pore system within the aggregates (pore surface fractal dimension) and decreased with pore volume and radius. Generally the maximum strength occurred at 64% of minerals content in the aggregates. Pure (100%) illite aggregate exhibited the highest strength.

The mechanical resistance of silt-mineral aggregates (excluding goethite) increased with decreasing mineral particles dimension and with an increase in the roughness of mineral particles surface (surface fractal dimension).

## Figures and Tables

**Table 1:** Basic properties of the materials used.

		Silt	Kaolinite	Montmorillonite	Zeolite	Illite	Goethite	Max SD%	Min SD%
*S_N2_*	m^2^·g^−1^	1.7	14.4	225.2	26.1	145.5	12.5	5.9 S	0.62 G
*d_particle_*	µm	15.0	0.66	0.58	0.51	0.49	0.39	44.4 K	2.56 G
ζ	mV	−45	−35.4	−51.5	−45.5	−23.2	12.1	10.7 G	1.35 M
*V_poreN2_*	mm^3^·g^−1^	0.7	6.6	119	16.5	52.5	40.5	4.9 I	0.18 M
*d_poreN2_*	nm	5.79	5.94	6.22	2.61	6.24	4.98	5.1 Z	0.39 M
*SPD*	g cm^−3^	2.70	2.62	2.52	2.33	2.74	4.11	0.18 G	0.03 S
*D_fracN2_*	-	2.25	2.55	2.58	2.62	2.70	2.63	1.6 S	0.7 K
*P*	%	0.2	1.7	23.1	3.7	12.6	14.3	-	-
*Q*	%	0.04	0.9	1.07	0.51	1.27	1.28	-	-

Parameters from up to down: *S_N2_*—specific surface area (from nitrogen adsorption), *d_particle_*—average particle diameter, ζ—zeta potential, *V_poreN2_*—volume of 10–30 nm micropores, *d_poreN2_*—average pore diameter, *SPD*—solid phase density, *D_fracN2_*—surface fractal dimension, *P*—volumetric porosity (fraction of pores), *Q*—average pore diameter to average particle diameter ratio. Two last columns show maximum and minimum standard deviations (SD) expressed as percents, followed by the first letter of the material for which particular value of SD occurred. The standard deviations are not presented for data calculated from average numerical values.

**Table 2:** Numerical values of *C* and *k* constants providing the best fits of the experimental data to the Langmuir-type equation (Equation (7)) for the dependencies of the maximum stress, σ_max_, and Young’s modulus, *E*, on minerals percentage presented in Figure 4.

Data	Constant *	Kaolinite	Monmorillonite	Zeolite	Illite	Goethite
σ_max_	*C_σ_*, MPa	3.0	4.1	6.2	11.6	1.1
	*k_σ_*	0.010	0.031	0.022	0.017	0.042
*E*	*C_E_*, MPa	272	362	424	393	55
	*k_E_*	0.013	0.064	0.043	0.116	0.771

* Each constant is abbreviated with a subscript being in accordance with the data for which it was derived.

**Table 3:** Mechanical and structural parameters characterizing the studied aggregates.

Material	MIN %	σ_max_ MPa	*E* MPa	*BD* gcm^−3^	*V_MIP,_*_max_ cm^3^g^−1^	*V_(BD)_* cm^3^g^−1^	*V*_ia_ cm^3^g^−1^	*R_MIPav_* µm	*D_fracMIP_* –	*PT* µm	*R*_ia_ µm
Silt		0.08	28.5	1.377	0.321	0.356	0.213	4.380	2.24	4.772	3.64
Kaolinite	2	0.10	26	1.389	0.317	0.350	0.167	3.956	3.07	4.336	2.79
	4	0.14	26.6	1.425	0.288	0.331	0.146	3.303	3.22	4.336	2.50
	8	0.27	30.7	1.465	0.252	0.312	0.207	2.252	3.51	3.633	1.59
	16	0.41	41.7	1.521	0.251	0.286	0.196	1.004	3.99	1.966	0.73
	32	0.66	70.9	1.700	0.217	0.215	0.175	0.248	3.33	0.444	0.18
	64	1.19	127	1.676	0.198	0.219	0.119	0.065	4.54	0.054	0.03
	100	0.92	110.6	1.533	0.268	0.271	0.139	0.061	4.68	0.054	0.03
Illite	2	0.32	73.7	1.406	0.320	0.341	0.185	4.036	2.87	4.336	2.97
	4	0.72	143	1.410	0.297	0.339	0.206	3.888	3.15	4.336	3.10
	8	1.33	195.7	1.487	0.270	0.303	0.114	2.280	3.41	2.176	1.22
	16	2.39	221.5	1.578	0.222	0.265	0.116	1.581	3.71	2.176	0.76
	32	4.42	318	1.715	0.191	0.215	0.160	0.594	4.04	1.777	0.43
	64	6.03	354	1.881	0.140	0.165	0.108	0.095	4.20	0.657	0.03
	100	8.86	510	1.860	0.138	0.173	0.056	0.013	4.51	0.003	0.00
Montmorillonite	2	0.11	31.1	1.389	0.307	0.349	0.209	3.756	2.55	4.336	2.97
	4	0.29	61.4	1.381	0.325	0.353	0.189	3.567	2.55	4.336	2.41
	8	0.72	90.2	1.434	0.304	0.325	0.205	2.449	2.37	3.633	1.70
	16	1.33	226.4	1.545	0.251	0.273	0.151	0.521	2.38	0.801	0.26
	32	2.40	251.7	1.536	0.254	0.272	0.162	0.219	3.38	0.540	0.10
	64	2.57	273.9	1.374	0.318	0.341	0.245	0.111	3.42	0.249	0.05
	100	2.11	236.2	1.016	0.503	0.512	0.362	0.066	3.43	0.205	0.05
Zeolite	2	0.11	36.1	1.377	0.332	0.355	0.178	4.241	2.56	4.336	3.18
	4	0.13	40.1	1.385	0.331	0.349	0.206	3.531	2.86	4.336	2.55
	8	0.55	72.3	1.431	0.325	0.324	0.265	2.608	3.16	4.336	2.16
	16	1.83	214.7	1.563	0.228	0.260	0.168	0.575	4.14	2.176	0.27
	32	2.96	255	1.726	0.177	0.190	0.097	0.089	4.16	0.079	0.05
	64	3.43	298.3	1.465	0.270	0.275	0.177	0.083	4.18	0.079	0.05
	100	2.53	247.9	1.096	0.472	0.483	0.361	0.070	4.66	0.079	0.05
Goethite	2	0.15	35.6	1.424	0.301	0.335	0.204	3.324	3.09	4.336	2.24
	4	0.19	40	1.457	0.303	0.321	0.179	3.006	2.97	3.939	1.81
	8	0.26	45.3	1.519	0.255	0.298	0.153	2.054	3.16	3.633	0.92
	16	0.41	51.6	1.629	0.244	0.264	0.186	0.705	2.87	2.176	0.37
	32	0.66	54.7	1.888	0.189	0.200	0.150	0.138	3.78	0.249	0.08
	64	0.82	121	1.717	0.289	0.293	0.148	0.093	3.84	0.071	0.06
	100	0.72	66.6	1.403	0.456	0.469	0.261	0.097	3.87	0.079	0.06
Max SD%	-	51.3	33.3	0.7	5.3	-	13.3	6.6	5.4	10.0	5.1
	-	G4	G4	S	G4	-	G2	Z2	S	G2	S
Min SD%	-	12.1	3.4	0.1	2.1	-	4.6	2.9	2.0	4.3	1.8
	-	I100	M64	I100	I100	-	Z64	M64	Z64	M64	I64

Parameters from left to right: σ_max_—maximum stress, *E*—Young’s modulus, *BD*—bulk density, *V_MIP,_*_max_—total pore volume (from mercury intrusion), *V_BD_*—pore volume (from bulk density), *V*_ia_—intraaggregate pore volume, *R_MIPav_*—average pore radius, *D_fracMIP_*—pore surface fractal dimension, *PT*—penetration threshold, *R*_ia_—average intraaggregate pore radius. The last rows show maximum and minimum standard deviations (SD) expressed as percents. Below them the first letter of the mineral component and its percentage in the aggregate for which particular value of SD occurred are shown. The standard deviations are not presented for data calculated from average numerical values.

**Table 4:** Coefficients of determination (R^2^) for linear fits between parameters characterizing aggregate strength and mineral particles properties. Only R^2^ values higher than 0.5 were taken into account. The sign preceding the R^2^ value is the sign of the slope of the respective linear fit.

Parameter	*S_N2_*	*d_particle_*	ζ	*V_poreN2_*	*d_poreN2_*	*D_fracN2_*	*P*	*Q*
*C_σ_*	-	−0.7	-	-	-	+0.99	-	-
k*_σ_*	0.53	-	−0.51	+0.69	-	-	+0.64	-
*C_E_*	-	−0.89	-	-	-	-	-	-
k*_E_*	-	−0.59	-	-	-	+0.80	-	-
σ_max_	-	−0.55	+0.56	-	-	+0.93	-	-
*E*	-	−0.69	-	-	-	+0.95	-	-

Strength parameters from top to bottom: shown in Table 2 values of *C* and k constants providing the best fits of the experimental data to the Langmuir-type equation for the dependencies of the maximum stress on minerals percentage (*C_σ_* and k_σ_) and for the dependencies of the Young’s modulus on minerals percentage (*C_E_* and k*_E_*). σ_max_—maximum stress for pure minerals aggregates; *E*—Young’s modulus for pure minerals aggregates. Properties of mineral particles (see Table 1) from left to right: *S_N2_*—specific surface area; *d_particle_*—particle diameter; ζ—zeta potential; *V_poreN2_*—volume of 2–30 nm pores; *d_poreN2_*—average pore diameter; *D_fracN2_*—surface fractal dimension; *P*—volumetric porosity, *Q*—pore diameter to particle diameter ratio.

## Data Availability

Data available from authors.

## Supplementary Materials

The following are available online at https://www.mdpi.com/article/10.3390/ma14164688/s1: Description of how the average stress-strain curves were derived. Illustrations of structural differences of artificial aggregates causing low reproducibility of stress-strain curves. Figure S1: Dependence of the compressive stress on strain for all studied 100% kaolinite and 32% illite aggregates. Figure S2: Representative 2D microtomography images of most different intrinsic structures of the replicates of the studied 100% kaolinite and 32% illite aggregates.
