# Supplementary material for: Structure and Strength of Artificial Soils Containing Monomineral Clay Fractions
Source: Materials (Basel). 2021 Aug 19;14(16):4688. doi: 10.3390/ma14164688 (PMC8400040; doi:10.3390/ma14164688)
Supplement: Supplementary file 1 [file materials-14-04688-s001.zip › materials-1330469-supplementary.pdf]

Supplementary Materials

Structure and Strength of Artificial Soils Containing Monomineral Clay Fractions.

It is known to everyone who has some experience with stress-strain measurements of granular materials that their breakage curves are not well reproducible. We frequently observed that at the same height to diameter ratio of cylindrical samples, the reproducibility decreases with the specimen volume decrease. Having limited amount of the minerals, in the experiments performed in this paper we used the largest aggregate sizes possible, however the reproducibility of their breakage curves was also rather low, as it is illustrated exemplary in Figure S1.

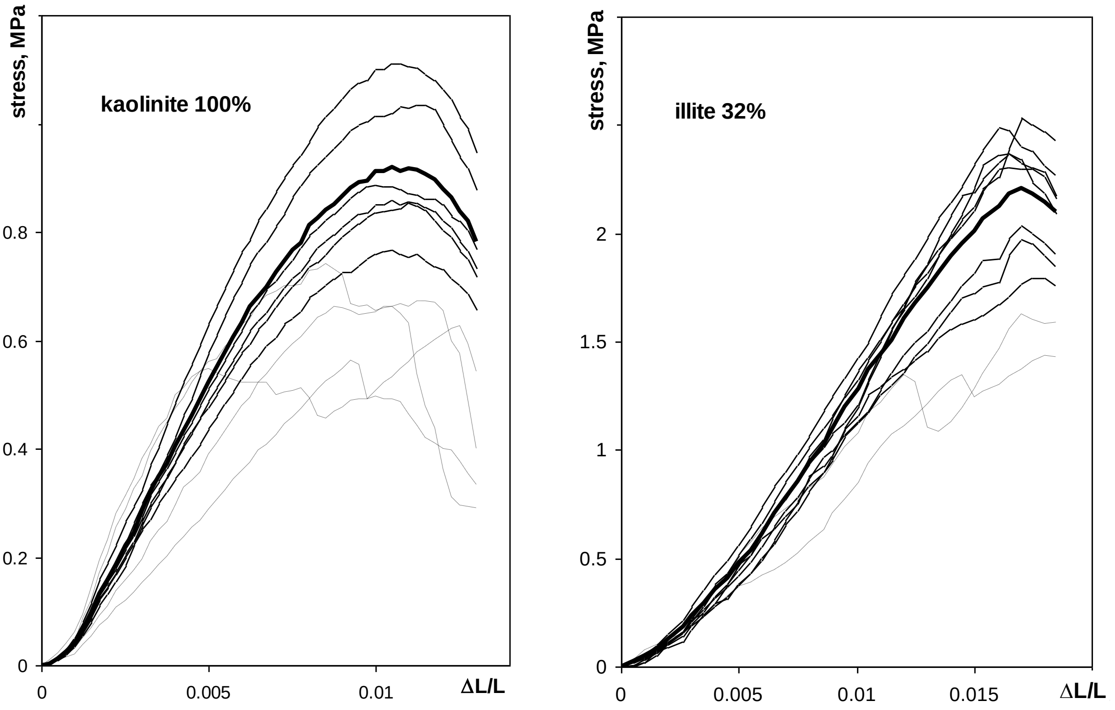

(**a**) (**b**)

Figure S1. Dependence of the compressive stress on strain for all studied 100% kaolinite (**b**) and 32% illite (**a**) aggregates. Thin solid curves are drawn for most similar ones. Thick solid line is drawn through the average values of the latter curves. Dashed curves illustrate aggregates differing markedly in strain-stress behavior.

Even for pure mineral aggregates the breakage curves do not satisfactorily coincide. One can observe that some curves (drawn with the dashed lines) are markedly differed from their (more similar) counterparts. We think that the lack of the curves coincidence as well as their larger differences are due to the presence of surface defects of the specimens and to larger structural discontinuities present within the aggregates. Even when the intrinsic structure of the aggregates is the same, the bottom and the top surfaces of the measured specimens may be not exactly parallel, and/or the geometry of the roughness of the bottom and/or the top surfaces differ between the specimens because the aggregates surfaces are not flat. If the piston contacts the

aggregate surface being rough and not exactly parallel to the piston, two breakage pathways are possible: either some additional force is needed to equalize the surface at the beginning of the compression that results in increasing of the registered breakage force, or some small cracks form initially at the specimen surface causing a spattering off some parts of the aggregate. As the result the piston/aggregate contact surface decreases and the registered breakage force decreases. Similar effect may occur if the vertical surface of the aggregate contains larger irregularities.

The next reason of breakage curves differences is that even during identical and very careful preparation procedure the same structure of the aggregates replicates may be not reproduced. To check it we applied X-ray computational microtomography (GE Nanotom S device, Frankfurt, Germany) to scan the replicates of the 100% kaolinite and 32% illite aggregates with the spatial resolution (voxel size) about 1 μm. Representative 2D cross-section images of most different replicates of the aggregates are shown in Figure S2. The scans were made for aggregates which remained after studies. We are aware that the best way would be to scan the structure of all studied aggregates prior to uniaxial compression, however due to extremely high costs and time consumption of the analysis we were not able to do it.

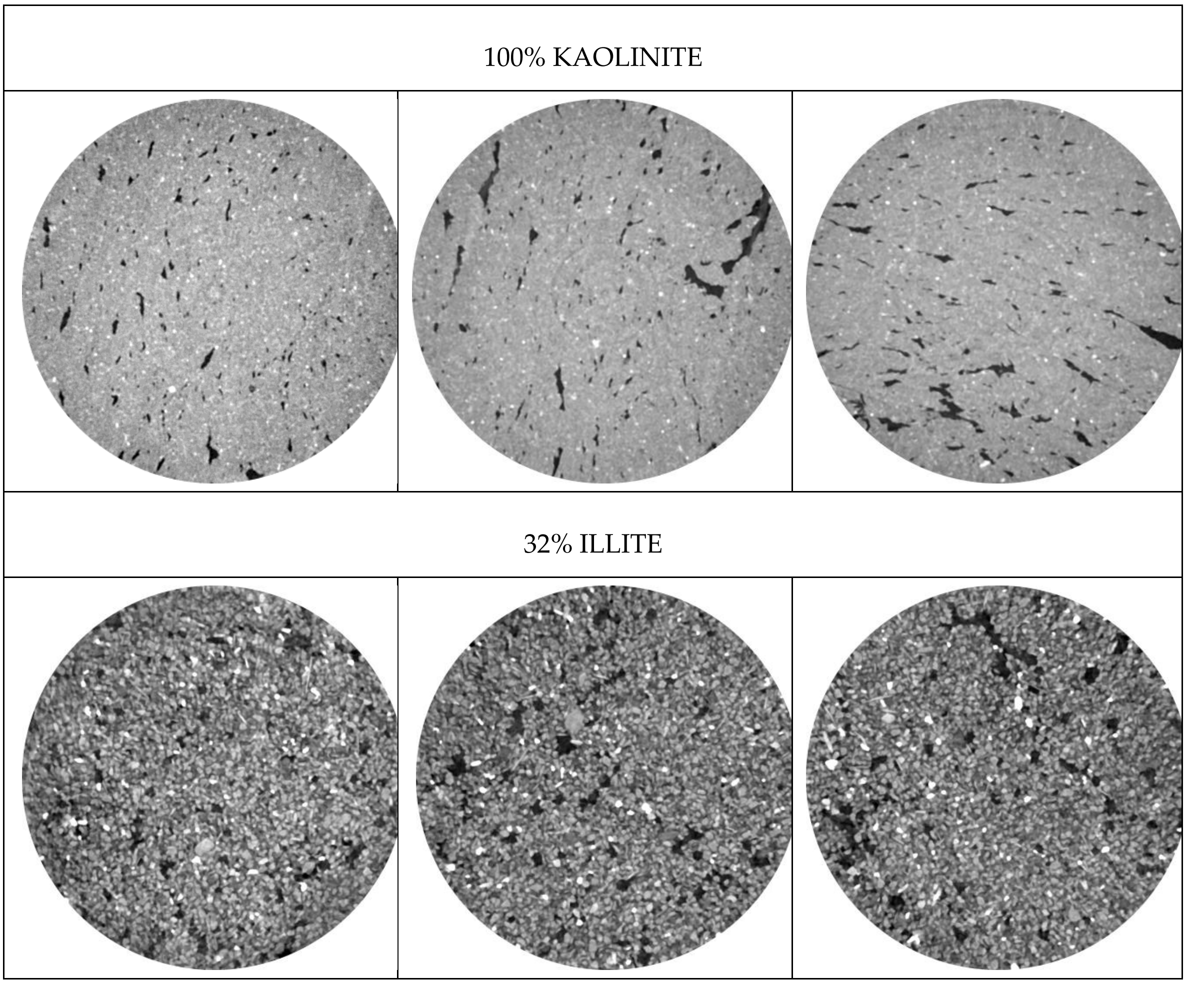

Figure S2. Representative 2D microtomography images of most different intrinsic structures of the replicates of the studied 100% kaolinite (up) and 32% illite (down) aggregates.

Black areas in the Figure S2 represent empty spaces (larger pores) formed occasionally within different aggregates. Differences in their

sizes and location induce differences in aggregates braking patterns. We think that larger pores formation is random and unavoidable in our aggregate preparation procedure. Possibly if high external pressure was applied during the preparation, the aggregates' intrinsic structure would be more uniform, however we wanted to approach more natural conditions of soil structure formation.

As it can be concluded from the above presented material, many factors are responsible for the location and the coincidence of the breakage curves presented in Figure S1. Since we could not find exact criteria for the appropriate curves selection for estimation of the strength parameters, we relied on visual analysis of the curves similarity and so we rejected the dashed curves from further considerations. We believe that the largest structural artifacts present within the aggregates or upon their surfaces were responsible for highly differing course of these curves.
